# Individual variation in the attribution of incentive salience to social cues

**DOI:** 10.1038/s41598-020-59378-5

**Published:** 2020-02-13

**Authors:** Christopher J. Fitzpatrick, Jonathan D. Morrow

**Affiliations:** 1 Neuroscience Graduate Program, University of Michigan, Ann Arbor, MI USA; 2 Department of Psychiatry, University of Michigan, Ann Arbor, MI USA

**Keywords:** Classical conditioning, Motivation, Reward, Social behaviour

## Abstract

Research on the attribution of incentive salience to drug cues has furthered our understanding of drug self-administration in animals and addiction in humans. The influence of social cues on drug-seeking behavior has garnered attention recently, but few studies have investigated how social cues gain incentive-motivational value. In the present study, a Pavlovian conditioned approach (PCA) procedure was used to identify rats that are more (sign-trackers; STs) or less (goal-trackers; GTs) prone to attribute incentive salience to food reward cues. In Experiment 1, a novel procedure employed social ‘peers’ to compare the tendency of STs and GTs to attribute incentive salience to social reward cues as well as form a social-conditioned place preference. In Experiment 2, social behavior of STs and GTs was compared using social interaction and choice tests. Finally, in Experiment 3, levels of plasma oxytocin were measured in STs and GTs seven days after the last PCA training session, because oxytocin is known to modulate the mesolimbic reward system and social behavior. Compared to GTs, STs attributed more incentive salience to social-related cues and exhibited prosocial behaviors (e.g., social-conditioned place preference, increased social interaction, and social novelty-seeking). No group differences were observed in plasma oxytocin levels. Taken together, these experiments demonstrate individual variation in the attribution of incentive salience to both food- and social-related cues, which has important implications for the pathophysiology of addiction.

## Introduction

Nearly every neuropsychiatric disorder involves alterations in social behaviors, and several disorders are characterized by abnormal processing of social cues^1^. Specifically, addiction pathophysiology involves the cooption and alteration of social behaviors and cue processing^2^. For example, social interactions within drug-taking contexts enhance drug-seeking behavior^3^, and social cues (e.g., people) can elicit similar reactivity and craving as drug-related cues^4,5^. Indeed, in a sample of adolescents, being around other peers was the greatest contributor to drug relapse (73%) regardless of whether the peers were using^6^. Tobacco and alcohol companies have long exploited social cues to promote drug consumption, incorporating such cues into 42% and 74% of their advertisements, respectively^7^. Interestingly, ‘social reinstatement’ of drug-seeking behavior has been recently demonstrated in rats (i.e., social ‘peers’ can serve as discriminative stimuli and increase drug-seeking behavior)^8^. However, there remains a lack of research linking the processing of social context and cues with addiction-like behaviors, or determining which individuals may be more or less susceptible to the reward-modifying aspects of social cues^9^.

Pavlovian conditioned approach (PCA) procedures have previously been used to investigate individual variation in the attribution of incentive-motivational value to food- and drug-related cues^10–13^. When environmental cues are paired with rewarding stimuli, some animals (goal-trackers; GTs) use the cue as a predictor of impending reward while others (sign-trackers; STs) attribute incentive-motivational value to the cues, making them reinforcing and capable of motivating behavior even in the absence of the reward itself^10,14^. For example, PCA behavior can be elicited by repeated presentation of a retractable lever that is immediately followed by delivery of a food reward in a different part of the testing chamber, regardless of the animal’s behavior. Under those circumstances, GTs will orient toward the lever and then quickly approach the location of impending food delivery, while STs will learn to approach and interact with the lever itself, only turning to retrieve the reward once the lever is retracted. In addition to increased cue-directed behavior, STs display several other behavioral traits that are believed to contribute to addiction-like behaviors, such as impulsivity^15^ and novelty-seeking behavior^16^.

Sign-tracking requires dopamine (DA) activity in the mesocorticolimbic system and is DA-dependent in the nucleus accumbens (NAc) core^17,18^. Because both nonsocial and social rewards and their cues are processed through the mesocorticolimbic DA system^19^, it is likely that STs would (1) sign-track to social-related cues and (2) show prosocial behaviors. Although social experience has been shown to modulate sign-tracking behavior^20,21^, sign-tracking to nonsexual social-related cues has not previously been investigated.

To address this, the present study measured individual variation in (1) the attribution of incentive salience to social-related cues and (2) social behaviors in rats. In Experiment 1, a novel procedure combining conditioned place and cue preference was used to measure sign-tracking to a social-related cue as well as social context in GTs and STs. In Experiment 2, social interaction and social choice tests were used to measure sociability and social novelty seeking in GTs and STs. Finally, in Experiment 3, plasma oxytocin (OXT) levels were measured seven days following PCA training under home-cage conditions in GTs, intermediate-responders (IRs; no bias in sign- and goal-tracking behaviors), and STs, and levels were correlated with PCA behavior. OXT was measured because it modulates DA release in response to social stimuli^22^ and regulates the salience of social cues^23^.

## Results

### Experiment 1: Sign-trackers but not goal-trackers attribute incentive-motivational value to a social-related cue, and they show social-conditioned place preference and aversion, respectively

**Figure 1:**
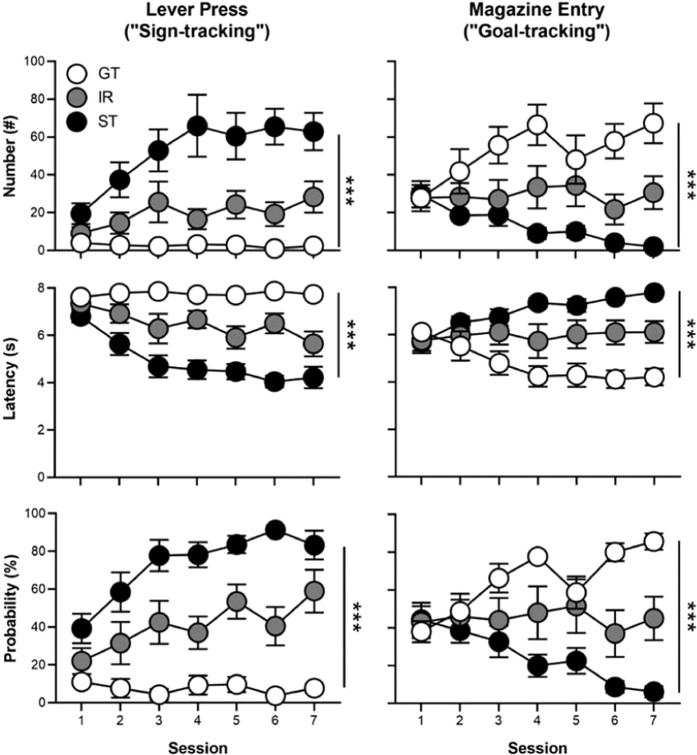
Rats underwent PCA training over seven daily sessions and were classified as sign-trackers (STs; n = 11), goal-trackers (GTs; n = 12), or intermediate-responders (IRs; n = 8) based on their lever press and magazine entry number, latency, and probability during Sessions 6 and 7. Only GTs and STs were used for subsequent testing (social conditioned place/cue preference procedure) in Experiment 1. Data are presented as mean ± S.E.M. ***p < 0.001.

**Figure 2:**
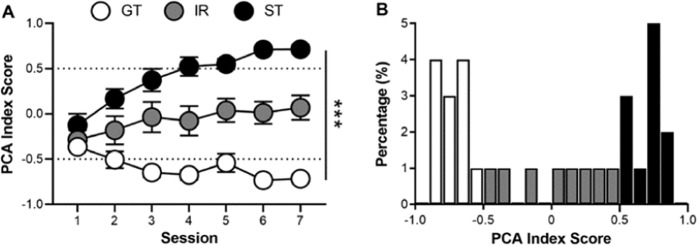
(**A**) Lever press and magazine entry number, latency, and probability were combined into a Pavlovian conditioned approach (PCA) index score for each session. On the PCA index, scores range between −1.0 (absolute goal-tracking) and +1.0 (absolute sign-tracking). The average PCA index scores from Sessions 6 and 7 were used to phenotype rats with the following cutoffs: goal-trackers (GTs, n = 12; × ≤ −0.5), intermediate-responders (IRs, n = 8; −0.5 < × < 0.5), and sign-trackers (STs, n = 11; × ≥ 0.5). (**B**) Rats were distributed across PCA index scores in a similar manner to previous reports^24,25^. Data are presented as mean ± S.E.M. ***p < 0.001.

Rats underwent seven daily sessions of PCA training and were classified as STs, IRs, and GTs based on their average PCA index scores over Sessions 6 and 7. (See Table S1 for a comparison between phenotypes of PCA variables and index scores averaged over Sessions 6 and 7.) Only STs and GTs were used for further testing. Figure 1 shows that STs, IRs, and GTs differed in their lever press number (effect of Phenotype: F_(2,35.75)_ = 15.07, p = 1.79 × 10^−5^), latency (effect of Phenotype: F_(2,35.41)_ = 31.36, p = 1.45 × 10^−8^), and probability (effect of Phenotype: F_(2,34.38)_ = 36.21, p = 3.46 × 10^−9^) as well as magazine entry number (effect of Phenotype: F_(2,44.44)_ = 14.09, p = 1.82 × 10^−5^), latency (effect of Phenotype: F_(2,34.12)_ = 8.87, p = 7.90 × 10^−4^), and probability (effect of Phenotype: F_(2,44.04)_ = 16.02, p = 5.93 × 10^−6^). Rats also differed on their PCA index score (Fig. 2A; effect of Phenotype: F_(2,36.52)_ = 46.86, p = 8.25 × 10^−11^), and distributions of phenotypes were similar to those that have been previously observed in larger samples (Fig. 2B)^24,25^.

**Figure 3:**
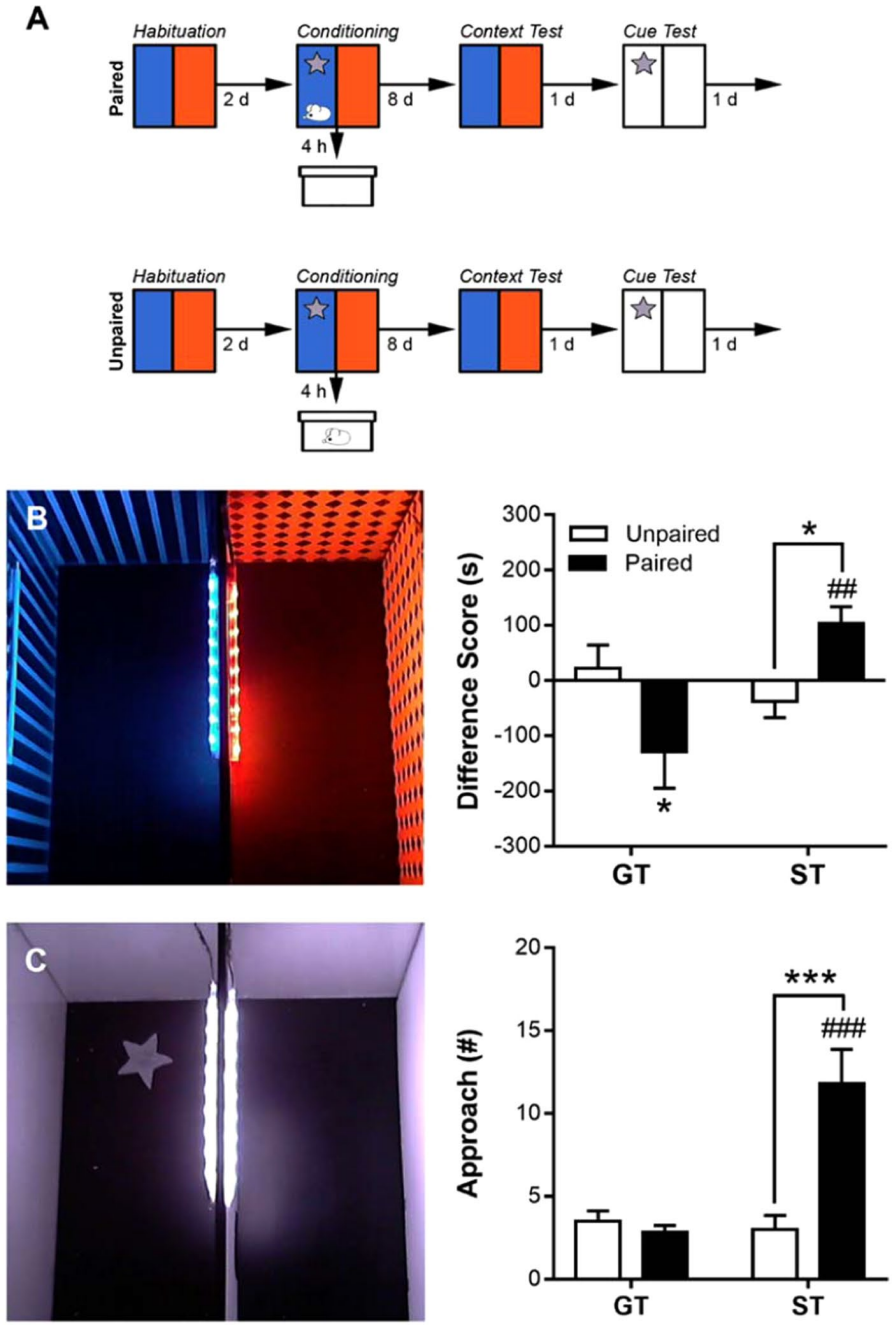
In Experiment 1, seven days after the last session of PCA training, sign-trackers (STs) and goal-trackers (GTs) underwent a (**A**) social conditioned place/cue preference procedure. Following two days of habituation and eight days of conditioning (with the context and a star-cue), paired and unpaired rats underwent a (**B**) context test and (**C**) cue test. A difference score (time spent in the social interaction chamber – time spent in the empty chamber) was calculated to measure preference for the context test. Approach to the star-cue was calculated to measure sign-tracking for the cue test. Data are presented as mean + S.E.M. *p < 0.05, ***p < 0.001, within-subjects comparison; ^##^p < 0.01, ^###^p < 0.001, between-subjects comparison.

During the social-conditioned place and cue preference test (Fig. 3A), each rat displayed a preference for one of the two chambers (i.e., spending 50% or more time in one chamber, averaged over the two daily habituation sessions); however, there was no difference between the time that GTs or STs spent in the preferred or non-preferred chambers (data not shown; interaction of Phenotype × Chamber: F_(31.68)_ = 0.008, p = 0.93). As mentioned in the Methods section, rats were conditioned in the nonpreferred chamber. Following eight daily sessions of conditioning (four conditioned and four nonconditioned), rats underwent a context and cue test. During the context test, paired groups conditioned more than unpaired groups, and STs and GTs differed in their place conditioning (Fig. 3B; interaction of Phenotype × Group: F_(1,19)_ = 10.39, p = 0.004). Post hoc comparisons revealed that paired STs established a place preference (p < 0.05) and unpaired STs did not; in addition, paired STs formed a place preference more than paired GTs (p < 0.001). Moreover, paired GTs, but not unpaired GTs, formed a place aversion (p < 0.05). During the cue test, paired groups approached (sign-tracked towards) the star-cue more than unpaired groups, and STs and GTs differed in their number of approaches (Fig. 3C; interaction of Phenotype x Group: F_(1,19)_ = 5.69, p = 3.01 × 10^−4^). Post hoc comparisons revealed that paired STs sign-tracked to the star-cue more than unpaired STs, paired GTs, and unpaired GTs (ps < 0.001).

### Experiment 2: Sign-trackers but not goal-trackers show social novelty-seeking behavior and increased social interaction

Rats underwent seven daily sessions of PCA training and were classified as STs, IRs, and GTs based on their average PCA index scores over Sessions 6 and 7. Only STs and GTs were used for further testing. STs and GTs differed in their lever press number (data not shown; effect of Phenotype: F_(2,18.34)_ = 14.07, p = 1.83 × 10^−4^), latency (effect of Phenotype: F_(2,18,87)_ = 15.23, p = 1.15 × 10^−4^), and probability (effect of Phenotype: F_(2,19.66)_ = 14.81, p = 1.19 × 10^−4^) as well as magazine entry number (effect of Phenotype: F_(2,26.53)_ = 14.08, p = 6.81 × 10^−5^), latency (effect of Phenotype: F_(2,26.50)_ = 18.48, p = 9.43 × 10^−6^), and probability (effect of Phenotype: F_(2,23.34)_ = 14.35, p = 8.62 × 10^−5^). Rats also differed on their PCA index score (data not shown; effect of Phenotype: F_(2,22.45)_ = 31.40, p = 3.13 × 10^−7^).

**Figure 4:**
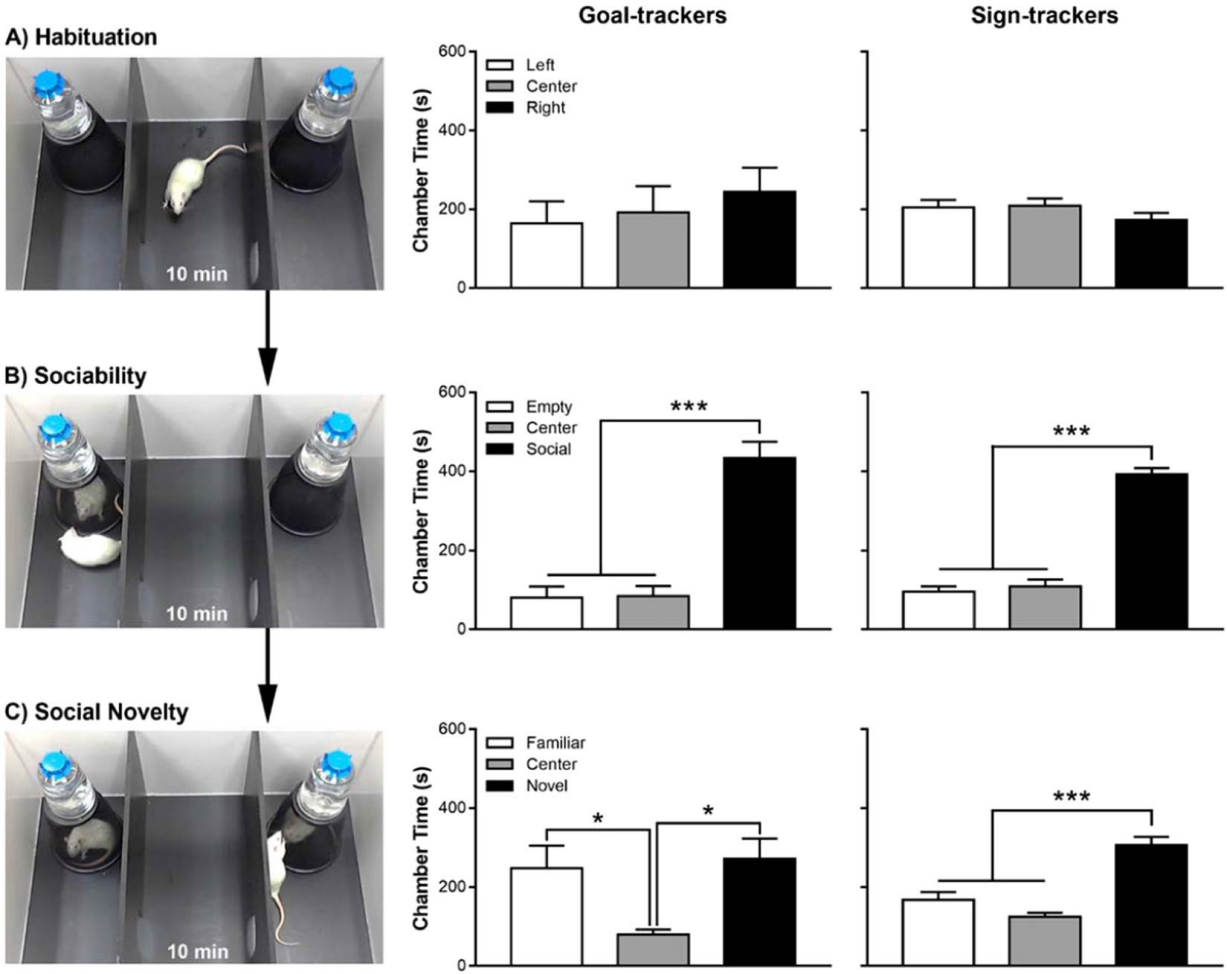
In Experiment 2, sign- and goal-trackers underwent a social choice test that consisted of three consecutive 10-min phases: (**A**) habituation (exposure to the test arena), (**B**) sociability (exposure to a partner rat), and (**C**) social novelty (simultaneous exposure to the now familiar partner rat and a novel partner rat). Data are presented as mean + S.E.M. *p < 0.05, ***p < 0.001.

Seven days following the last session of PCA training, rats underwent a social choice test, which consisted of three consecutive, 10-min phases: habituation, sociability, and social novelty. During the habituation phase, neither GTs (Fig. 4A; effect of Chamber: F_(2,12)_ = 0.43, p = 0.66) nor STs (effect of Chamber: F_(2,18)_ = 1.38, p = 0.28) showed an initial preference for any of the chambers. Next, during the sociability phase, GTs (Fig. 4B; effect of Chamber: F_(2,12)_ = 39.28, p = 5.42 × 10^−6^) and STs (effect of Chamber: F_(2,18)_ = 109.84, p = 8.19 × 10^−11^) both preferred the chamber containing a partner rat. Post hoc comparisons revealed that both STs and GTs spent more time in the chamber containing a partner than the center (p < 0.001) and empty (p < 0.001) chambers. Finally, during the social novelty phase, STs (Fig. 4C; effect of Chamber: F_(2,18)_ = 0.14, p = 2.55 × 10^−6^) but not GTs (effect of Chamber: F_(2,12)_ = 0.14, p = 0.87), showed social novelty-seeking behavior. Post hoc comparisons revealed that STs spent more time in the chamber containing a novel partner compared to the center chamber (p < 0.001) and chamber containing the familiar rat (p < 0.001). In addition, post hoc comparisons revealed that GTs spent more time in the chambers containing rats compared to the center chamber (p < 0.01); however, they did not discriminate between the chambers containing the familiar and novel rats (p > 0.05).

**Figure 5:**
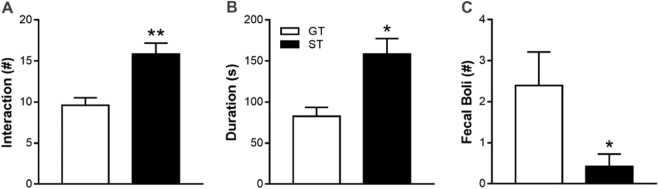
In Experiment 2, sign-trackers (STs) and goal-trackers (GTs) underwent a social interaction test, and the (**A**) number and (**B**) duration of social interactions as well as (**C**) fecal boli (a measure of social anxiety-like behavior) were measured. Data are presented as mean + S.E.M. *p < 0.05, **p < 0.01.

Next, seven days after the social choice test, rats underwent a social interaction test during which the number and durations of active social interactions as well as fecal boli were measured. STs, compared to GTs, performed more social interactions (Fig. 5A; effect of Phenotype: t_10_ = −3.52, p = 0.006) and for longer durations (Fig. 5B; effect of Phenotype: t_10_ = −3.09, p = 0.011). In addition, STs defecated less than GTs during the social interaction test (Fig. 5C; effect of Phenotype: t_10_ = 2.59, p = 0.027).

### Experiment 3: Plasma OXT levels are not different between GTs, IRs, and STs

**Figure 6:**
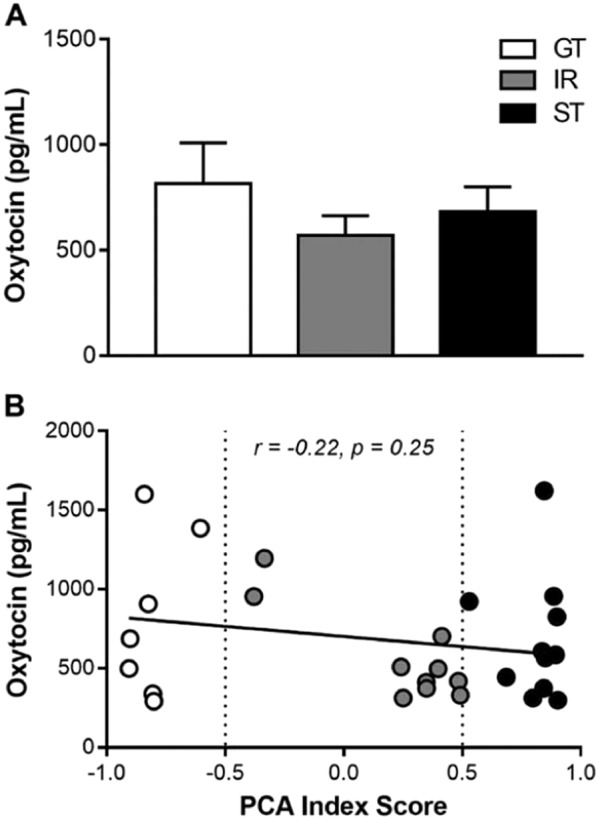
In Experiment 3, plasma oxytocin samples were collected from the home cage seven days after the last Pavlovian conditioned approach (PCA) training session. (**A**) Plasma oxytocin samples were compared between goal-trackers (GTs), intermediate-responders (IRs), and sign-trackers (STs). In addition, (**B**) plasma oxytocin levels were correlated with PCA index scores (averaged between Sessions 6 and 7). Data are presented as mean + S.E.M.

Rats underwent seven daily sessions of PCA training and were classified as STs, IRs, and GTs based upon the PCA index scores from Sessions 7. One rat was excluded for not learning any conditioned response (i.e., Session 7 PCA index score = 0). STs, IRs, and GTs differed in their lever press number (data not shown; effect of Phenotype: F_(2,36.16)_ = 41.28, p = 4.64 × 10^−10^), latency (effect of Phenotype: F_(2,37.67)_ = 42.53, p = 2.18 × 10^−10^), and probability (effect of Phenotype: F_(2,37.86)_ = 53.36, p = 9.65 × 10^−12^) as well as magazine entry number (effect of Phenotype: F_(2,39.32)_ = 19.47, p = 1.33 × 10^−6^), latency (effect of Phenotype: F_(2,37.98)_ = 16.85, p = 5.78 × 10^−6^), and probability (effect of Phenotype: F_(2,36.40)_ = 13.13, p = 5.08 × 10^−5^). Rats also differed on their PCA index score (data not shown; effect of Phenotype: F_(2,36.01)_ = 41.81, p = 4.10 × 10^−10^). Seven days following the last session of PCA training, rats were removed from their home cages and plasma OXT samples were collected. GTs, IRs, and STs did not differ in their levels of OXT (Fig. 6A*;* effect of Phenotype: F_(2,25)_ = 0.81, p = 0.46). In addition, levels of OXT did not correlate with PCA index scores (Fig. 6B; r = −0.22, p = 0.25).

## Discussion

In the present study, we demonstrated convergent individual variation in the attribution of incentive-motivational value to both a food- and social-related cue. In other words, rats that sign-track towards a food-related cue also sign-track towards a social-related cue. In addition, we demonstrated that STs display prosocial behaviors (e.g., social-conditioned place preference, social novelty-seeking and increased social interaction) while GTs exhibit antisocial behaviors (e.g., social-conditioned place aversion, absent social novelty-seeking, and anxiety-like behavior during social interaction). Finally, PCA phenotypes did not differ in plasma levels of OXT measured seven days after PCA training under home-cage conditions, and OXT did not correlate with PCA index scores. Taken together, these results show that (1) social-related cues can promote sign-tracking behavior, (2) the attribution of incentive-motivational value to reward cues contributes to individual variation in social behaviors, and (3) OXT levels do not appear to differ between PCA phenotypes.

The present study is the first to show sign-tracking to a nonsexual, social-related cue. Previously, it has been demonstrated that Japanese quail sign-track towards sexual reward-related cues^26^. Humans also appear to sign-track toward sexual cues^27^. Despite their similarities, however, sexual and nonsexual social rewards have distinct valences and motivational properties^28^. Also, our results demonstrate that STs can attribute incentive-motivational value to more than one conditioned stimulus (e.g., a lever predicting food delivery and a star predicting social interaction), and behaviors related to sign-tracking (increased novelty-seeking and conditioned place preference) are consistent across different stimuli and procedures^29,30^. Finally, this study is the first to demonstrate individual variation in social behaviors in an outbred rodent population. Previously, it has been demonstrated that there are strain differences in conditioned place preference/aversion, social interaction, and social novelty-seeking in rodents^31–34^; however, all these studies were performed in inbred populations.

Sign-tracking to food and drug-related cues requires DA signaling in the NAc^17,18,35^. Because the NAc encodes reward and motivation for social-related cues in an analogous manner to food- and drug-related cues, it is highly likely that sign-tracking to social-related cues—like drug- and food-related cues—is DA-dependent in the NAc^36^. In addition, DA signaling in the NAc encodes and is sufficient to regulate social behavior^37^, suggesting that individual differences in NAc DA signaling may underlie differences in social behavior observed in the present study. For example, increased DA signaling in the NAc of STs during social experiences can explain the formation of social-conditioned place preference^38^, increased social interaction^39^, and social novelty-seeking^40^. Conversely, decreased DA signaling in the NAc of GTs during social experiences might also explain the formation of social conditioned place aversion^38^ and decreased social interaction^39^.

OXT is a neuropeptide that has a documented role in promoting social behavior and reward by activating dopaminergic pathways in the mesocorticolimbic reward system in response to social experiences and cues^36,41,42^. For example, OXT promotes attention to and processing of social cues^43,44^, enhances social interaction^45^, facilitates social discrimination and novelty-seeking^46–48^, produces conditioned place preference^49^, and is necessary for social memory formation^50^. In addition, OXT activates a social learning circuit—the prefrontal cortex, lateral septum, amygdala, ventral hippocampus, NAc, and ventral tegmental area^51–53^—that overlaps with the “motive circuit” underlying sign-tracking behavior^13,54^.

In the present study, plasma levels of OXT, collected seven days after PCA training, did not differ between GTs, IRs, and STs; in addition, plasma OXT levels did not correlate with PCA index scores. In future studies, plasma OXT levels (or central expression of OXT in the brain) could be measured at different time points such as a time before any PCA training has been conducted, or after social interaction or the presentation of social-related cues. It is at least possible that the PCA procedure has long-lasting effects on OXT levels that persist even past the seven-day resting period employed in this experiment. It is also possible that phenotypic differences in OXT release are only detectable immediately following exposure to social stimuli and cues, for example because the phenotypes differ specifically in the responsiveness of the OXT system to social stimuli.

In addition to OXT, other neurohormones might contribute to social sign-tracking and individual differences in social behaviors. First, vasopressin signaling modulates social interaction^55^, social recognition^56,57^, and even avoidance of social cues under certain circumstances^58^. Because vasopressin can be released in brain regions activated during the expression of sign-tracking behavior (e.g., periventricular nucleus of the hypothalamus, septum, and amygdala)^13,54^, it is possible that vasopressin signaling regulates sign-tracking behavior and individual differences in social behavior^59^. Future studies should measure peripheral and central levels of vasopressin in STs, IRs, and GTs during social interaction and/or presentation of social cues.

Second, dynorphin signaling through kappa opioid receptors (KORs) has been implicated in the regulation of DA signaling, negative affective states, cue-directed behavior, and social memory formation^60–62^. Relevant to the present study, KOR signaling in DA terminals originating from the VTA and terminating in the NAc contributes to conditioned place aversion, social avoidance, and decreased social interaction in rodents^63–66^. Because KOR agonism decreases DA release in the NAc^67^ and produces negative affective states, it is possible that increased KOR signaling is responsible for the antisocial behaviors in GTs (e.g., conditioned place aversion, decreased social interaction, lack of social novelty-seeking, and anxiety-like behavior during social interaction). Conversely, decreased KOR signaling surrounding social experiences might permit STs to attribute incentive-motivational value to social-related cues. Although dynorphin and vasopressin expression were not investigated in the present study, their potential role in sign-tracking to social-related cues and individual differences in social behavior merits further investigation.

The results from the present study have important implications for the treatment of addiction, because social cues and context are believed to contribute to the pathophysiology of drug addiction^68^. For example, social cues^69,70^ and contexts^71–73^ impact reward anticipation and craving. Furthermore, social pressure from peers can induce cravings and promote drug use^74,75^, and drug users show greater striatal activation during peer conformity to social information^76,77^. Conversely, social rewards can also acquire enough motivational value to successfully compete with addictive substances and promote voluntary abstinence from drug self-administration^78^. One of the most effective psychotherapies for addiction is coping and social skills training, which teaches social strategies for navigating social interactions, addressing interpersonal problems, and managing craving in response to social/drug-related cues and contexts^79^. In addition, new behavioral interventions for addicted patients are centered around social networks^80^, which are an important modulator of drug use and relapse^81^. A better understanding of sign-tracking to social-related cues and individual variation in social behaviors can aid the development and refinement of therapeutic interventions aimed at restoring a healthy balance between prosocial behaviors and the pursuit of nonsocial rewards in patients with addiction and other related disorders.

## Methods

### Animals

Adult male Sprague Dawley rats (250–300 g) were purchased from Charles River Laboratories. Rats were pair-housed and maintained on a 12 h light/dark cycle. Standard rodent chow and water were available *ad libitum*. Rats were acclimated to the housing colony for one week and handled for two days before any procedures commenced. All procedures were approved by the University of Michigan Institutional Animal Care and Use Committee (University of Michigan; Ann Arbor, MI), and all methods were performed in accordance with the relevant guidelines and regulations.

### Apparatus

Modular conditioning chambers (24.1 cm width × 20.5 cm depth × 29.2 cm height; MED Associates, Inc.; St. Albans, VT) were used for Pavlovian conditioned approach training, configured as previously described^82^. Briefly, each chamber was contained within a sound-attenuating cubicle equipped with a ventilation fan to provide ambient white noise. For Pavlovian conditioning, chambers were equipped with a pellet magazine on the front wall, an illuminated retractable lever (counterbalanced on the left or right of the pellet magazine), and a red house light on the back wall opposite to the pellet magazine. When inserted into the chamber, the retractable lever was illuminated by an LED light within the lever housing. A pellet dispenser delivered banana-flavored food pellets into the pellet magazine, and an infrared sensor inside the pellet magazine detected head entries.

A three-chambered apparatus (60 cm width × 90 cm length × 34 cm height; Formtech Plastics; Oak Park, MI) was used for social behaviors. Each chamber (60 cm width × 30 cm length × 34 cm height) consisted of closed-cell foamboard floors and walls (matte black polyvinyl chloride) and was connected by foamboard dividers with archways (10 cm width × 12 cm height at apex) to allow access between chambers. For the social choice test, the dividers remained in the apparatus. For the social interaction text, all dividers were removed, creating a single open arena (60 cm width × 90 cm length). For the social conditioned place/cue test, two chambers were used, and the third chamber was blocked.

### Pavlovian conditioned approach: procedure

Pavlovian conditioned approach was performed as previously described^82,83^. For two days prior to pretraining, rats were familiarized with banana-flavored food pellets (45 mg; Bioserv; Frenchtown, NJ) in their home cages (25 pellets/cage). Aside from the banana flavoring, the pellets closely mirror the nutritional value of standard rat chow (52% carbohydrate, 20.2% protein, 11.5% fiber, 6.3% fat). Twenty-four hours later, rats were placed into the operant chambers and underwent one pretraining session during which the red house-light remained on, but the lever was retracted. Fifty food pellets were delivered on a variable interval (VI) 30 schedule (i.e., one food pellet was delivered on average every 30 s, but actual delivery varied between 0–60 s). All rats consumed all of the food pellets by the end of the pretraining session. Twenty-four hours later, rats underwent daily PCA training sessions over seven days. Each trial during a test session consisted of extension of the illuminated lever (i.e., conditioned stimulus) into the chamber for 8 s on a VI 90 schedule (i.e., one food pellet was delivered on average every 90 s, but actual delivery varied between 30–150 s). Retraction of the lever was immediately followed by the response-independent delivery of one food pellet (i.e., unconditioned stimulus) into the pellet magazine. Each test session consisted of 25 trials of conditioned/unconditioned stimulus pairings, resulting in a total session length of approximately 40 min. All rats consumed all the food pellets that were delivered, and no rats were excluded from further behavioral testing for failing to consume pellets during training.

### Experiment 1: Social conditioned place/cue preference test

Rats underwent seven daily PCA training sessions to screen rats as STs (n = 11), GTs (n = 12), and IRs (n = 8). Only STs and GTs were used for further testing. IRs were used as partner rats during conditioning. Seven days after the last session of PCA training, rats were divided into Paired and Unpaired groups (ST/Paired, n = 5; ST/Unpaired, n = 6; GT/Paired, n = 6; GT/Unpaired, n = 6) and underwent a novel social conditioned place/cue preference procedure. The two chambers of the apparatus were differentiated using visual and olfactory cues. The left chamber had a white background with vertical black stripes, was illuminated internally by blue light (DIODER LED light strips; IKEA, Conshohocken, PA; wrapped in blue acetate film), and was wiped with a 1% almond extract solution (Context A). The right chamber had a white background with black diamonds, was illuminated by yellow light (DIODER LED light strips; wrapped in yellow acetate film), and was wiped with a 0.5% lemon extract solution (Context B). Blue and yellow light was selected, because it has previously been shown that rats can visually discriminate between these colors^84^. Moreover, the internal illumination prevented shadows, which is important as rats tend to remain immobile in shadowed areas of test arenas^85^. In addition, almond and lemon extracts were used, because rats do not show a preference or aversion to these neutral, distinguishable odors^86^.

Training consisted of two daily habituation sessions, eight daily conditioning sessions, and two daily test sessions. All sessions were 10 min in length. During habituation sessions, rats were exposed to the apparatus and allowed to freely explore between the two chambers. The average time spent in each chamber over the two habituation sessions was calculated, and the less preferred chamber was selected as the social-paired chamber. During conditioning sessions, a silver-painted star (cut from close-cell, polyvinyl chloride foamboard) was used as a discrete cue (star-cue) and placed in the social- but not empty-paired chamber. Use of the star-cue was loosely adapted from a previous study that demonstrated sign-tracking to an ethanol-paired star-cue in mice^87^. During conditioning sessions, rats in the paired group were placed on alternating days into an empty chamber or a chamber containing a rat, and access to the unused chamber was blocked. At the start of conditioning, partner rats were weight-matched to subject rats, and subject/partner rats never came from the same home cage. In addition, rats from the same home cage were never used as partners, and a novel partner rat was used on each conditioning day to prevent decreased social investigation over repeated pairings^88^. In the unpaired group, rats were placed on alternating days into one of two empty chambers. Four hours after the sessions, during which paired rats received social interaction and unpaired rats received nothing, paired rats were placed alone in a novel home cage, and unpaired rats received social interaction in a novel home cage. Twenty-four hours after the last conditioning session, rats underwent context and cue tests. During the context test, the star-cue was removed, and rats could freely explore both chambers (Context A and B). During the cue test, the star-cue was placed in the social-paired chamber, but the context in both chambers was changed to a third, neutral context: white walls, illumination by white light, and cleaning with a 70% ethanol solution (Context C). Like the context test, rats could freely explore both chambers.

### Experiment 2: Social choice and social interaction tests

A second cohort of rats underwent seven days of PCA training to screen rats for STs (n = 7), GTs (n = 5), and IRs (n = 8). Only STs and GTs were used for further testing. IRs were used as partner rats during both tests. Seven days after the last session of PCA training, rats underwent a social choice test. The social choice test was conducted in a three-chambered apparatus and consisted of three 10-min phases: habituation, sociability, and social novelty. Each phase immediately followed the previous phase. For all phases, subject rats were initially placed into the middle chamber on the side farthest from the doorways, and chambers were cleaned with a 70% ethanol solution between phases. During the habituation phase, the three-chambered apparatus contained two wire-mesh baskets (23 cm height, 13 cm top diameter, 22 cm bottom diameter) in the left and right chambers. Behavior was recorded during this phase, and a chamber preference was determined for each individual subject rat. During the sociability phase, the non-preferred chamber contained a partner rat inside a wire-mesh basket, and the preferred side contained an empty wire-mesh basket. Placement of partner rats was counterbalanced between left and right chambers. During the social novelty phase, the previously empty basket contained a novel rat, and the now familiar rat was contained within the other basket. During all phases, partner rats were always from different home cages than subject rats.

Seven days after the last phase of the social choice test, rats underwent a social interaction test, and novel, weight-matched partner rats were used. Partner rats were placed first in an open arena (60 cm width × 90 cm length), followed shortly after by placement of subject rats in the opposite corner. Similar to Experiment 1, partner rats were weight-matched to subject rats, and subject/partner rats never came from the same home cage. Social behaviors and fecal boli from the subject rat (a measure of anxiety-like behavior) were recorded by video camera for 10 min, and after the end of each session the arena was cleaned with a 70% ethanol solution.

### Experiment 3: Measurement of plasma OXT levels in GTs, IRs, and STs

A third cohort of rats underwent seven daily PCA training sessions to screen rats as GTs (n = 7), IRs (n = 10), and STs (n = 11). Seven days after the last PCA training session, rats were removed from their home cages to measure plasma OXT levels. Rats were rapidly decapitated, trunk blood was collected into chilled, EDTA-coated tubes (10 mL BD Vacutainer® tubes; Becton, Dickinson and Company; Franklin Lakes, NJ), and plasma was separated by centrifugation at 1,600 g for 15 min at 4 °C. Next, plasma was aliquoted and stored at −80 °C within 5 min of collection. An enzyme-linked immunosorbent assay (ELISA; ENZO Life Sciences, Inc.; Farmingdale, NY) was used to quantify plasma OXT levels. Unextracted samples were diluted 1:4 in assay buffer as previously described^89,90^. The dilution has been shown to reliably fit unextracted samples on a standard curve. Coefficient of variance was used as a cut-off, and three plasma samples were excluded for having a coefficient of variance higher than 20% (ST = 2, IR = 1).

### Statistical analysis

PCA behavior was scored using an index that combines the number, latency, and probability of lever presses (sign-tracking) and magazine entries (goal-tracking) during presentations of the conditioned stimulus within a session^25,82^. Briefly, we averaged together the response bias (i.e., number of lever presses and magazine entries for a session; [lever presses − magazine entries]/[lever presses + magazine entries]), latency score (i.e., average latency to perform a lever press or magazine entry during a session; [magazine entry latency − lever press latency]/8), and probability difference (i.e., proportion of lever presses or magazine entries; lever press probability − magazine entry probability). The index scores behavior from +1.0 (absolute sign-tracking) to −1.0 (absolute goal-tracking), with 0 representing no bias. Rats were classified using the following cutoffs: STs (x ≥ 0.5), IRs (−0.5 < × < 0.5), and GTs (x ≤ −0.5). Rats were classified by the average PCA index scores from Sessions 6 and 7.

In Experiments 1 and 2, social behaviors were recorded by camera and scored manually. For the social conditioned place/cue procedure and social choice test in Experiment 1, time spent in chambers was measured when the nose-point of a rat crossed through a doorway into another chamber. For the context test in Experiment 1, difference scores (time spent in the social interaction chamber – time spent in the empty chamber) were calculated to measure chamber preference. For the cue test, approach to the star-cue was scored when the rat either touched, sniffed, or gnawed on it. In Experiment 2, during the social interaction test, the number and duration of active social interactions were measured, including sniffing, grooming, following, mounting, wrestling, jumping on, and crawling over/under the partner rat^91^. In addition, recorded videos of the social interaction test were scored for fecal boli from the subject rat as a measure of anxiety-like behavior.

SPSS (Version 24; IBM, Inc.) was used for all statistical analysis. Across PCA training sessions, lever press and magazine entry number, latency, and probability were analyzed using a linear mixed model with a covariance structure, selected using Akaike’s information criterion (i.e., the lowest number criterion represents the highest quality statistical model using a given covariance structure). In Experiment 1, for the social conditioned place/cue tests, chamber time was analyzed using two-way analysis of variance (ANOVA) with Phenotype (GT and ST) and Group (Unpaired and Unpaired) as factors. In Experiment 2, during the social choice test, chamber time in each phase was analyzed using a two-way ANOVA with Phenotype (GT and ST) and Chamber (Left and Right) as factors. For the social interaction test, number and duration of social interactions as well as number of fecal boli were analyzed using independent samples t-tests with Phenotype (GT and ST) as a factor. In Experiment 3, OXT levels were analyzed using a one-way ANOVA with Phenotype (GT, IR, and ST) as a factor. Correlations were performed using Pearson’s r. With a significant ANOVA, multiple comparisons were performed using Fisher’s Least Significant Difference (LSD) post hoc test.

## Supplementary information

Supplementary information.

## Data Availability

The datasets analyzed during the current study are available from the corresponding author on reasonable request.
