# Supplementary material for: Individual variation in the attribution of incentive salience to social cues
Source: Sci Rep. 2020 Feb 13;10:2583. doi: 10.1038/s41598-020-59378-5 (PMC7018846; doi:10.1038/s41598-020-59378-5)
Supplement: Supplementary file 1 — Supplementary information. [file 41598_2020_59378_MOESM1_ESM.docx]

**Supplemental Information**

**Individual variation in the attribution of incentive salience to social cues**

^1^Christopher J. Fitzpatrick and ^1,2^Jonathan D. Morrow

^1^Neuroscience Graduate Program, University of Michigan, Ann Arbor, MI, USA

^2^Department of Psychiatry, University of Michigan, Ann Arbor, MI, USA

Corresponding Author:

Jonathan D. Morrow

Biomedical Sciences Research Building

109 Zina Pitcher Pl, Room 5047

Ann Arbor, MI 48109-5720

[jonmorro@umich.edu](mailto:jonmorro@umich.edu)

Keywords: Pavlovian conditioned approach; sign-tracking; reward; learning; oxytocin

**Supplemental Table S1.** Average lever press and magazine entry number (#), latency (s), and probability (%) across Pavlovian conditioned approach (PCA) Sessions 6 and 7 in sign-trackers (STs), intermediate-responders (IRs), and goal-trackers (GTs).

| **Phenotype** | **LP(#)** | **LP(s)** | **LP(%)** | **ME(#)** | **ME(s)** | **ME(%)** | **PCA Index** |
| --- | --- | --- | --- | --- | --- | --- | --- |
| GT (n = 12) | 1.75 ± 0.61 | 7.81 ± 0.06 | 5.67 ± 1.92 | 62.54 ± 9.02 | 4.17 ± 0.35 | 82.83 ± 4.43 | -0.72 ± 0.03 |
| IR (n = 8) | 23.75 ± 7.15 | 6.08 ± 0.46 | 49.75 ± 10.47 | 21.19 ± 7.07 | 6.11 ± 0.47 | 41.0 ± 10.82 | 0.04 ± 0.12 |
| ST (n = 1) | 64.18 ± 8.99 | 4.14 ± 0.26 | 87.27 ± 3.96 | 3.05 ± 1.09 | 7.68 ± 0.12 | 7.45 ± 2.87 | 0.71 ± 0.03 |
| *F-statistic* | *26.96* | *53.54* | *64.35* | *21.17* | *32.8* | *45.66* | *166.49* |
| *t-statistic* | *2.97 x 10^-7^* | *2.71 x 10^-10^* | *3.38 x 10^-11^* | *2.51 x 10^-6^* | *4.6 x 10^-8^* | *1.54 x 10^-9^* | *2.86 x 10^-16^* |
